# The Impact of Outpatient Prenatal Care Visitor Restrictions on Pregnant Patients and Partners During the COVID-19 Pandemic

**DOI:** 10.1089/whr.2022.0031

**Published:** 2022-08-04

**Authors:** Christina Collart, Caitlin Craighead, Susannah Rose, Richard Frankel, Brownsyne Tucker Edmonds, Uma Perni, Edward K. Chien, Marissa Coleridge, Angela Ranzini, Ruth M. Farrell

**Affiliations:** ^1^ Subspecialty Care for Women's Health, OB/GYN and Women's Health Institute, Cleveland Clinic, Cleveland, Ohio, USA.; ^2^ Center for Patient Experience, Cleveland Clinic, Cleveland, Ohio, USA.; ^3^ Department of the Lerner Research Institute, Cleveland Clinic Lerner College of Medicine, Cleveland, Ohio, USA.; ^4^ Department of OB/GYN, Indiana University, Indianapolis, Indiana, USA.; ^5^ Center for Personalized Genetic Healthcare, Department of Genomic Medicine Institute, Cleveland Clinic, Cleveland, Ohio, USA.; ^6^ Department of OB/GYN, MetroHealth Medical Center, Cleveland, Ohio, USA.; ^7^ Center for Bioethics, Cleveland Clinic, Cleveland, Ohio, USA.

**Keywords:** prenatal care, shared decision-making, visitor restriction, patient experience, COVID-19, mental health

## Abstract

**Introduction:**

During the early months of the COVID-19 pandemic, several health care facilities enacted visitor restrictions to help reduce the spread of SARS-CoV-2 among patients, front-line workers in health care systems, and communities. The impact and burden of policy updates on visitor restrictions put forth by the COVID-19 pandemic can be seen on patients and families, most often in the acute care setting and skilled nursing facilities. Yet, the effects of visitor restrictions in the prenatal care setting were unknown. We conducted a study to investigate the impact of these policies on pregnant patients who received outpatient prenatal care.

**Methods:**

We conducted a qualitative study to explore pregnant patients' experiences with prenatal health care delivery between May and July 2020. In-depth interviews were conducted with pregnant patients in the first and second trimester of pregnancy, who received their prenatal care at the onset of the pandemic in the United States.

**Results:**

Participants noted increased maternal concern, anxiety, and mental health concerns stemming from the lack of in-person partner support. They noted disappointment and lost experiences for the patient during pregnancy, seeking support from her partner during pregnancy, experiences felt to be critical for postpartum health and wellbeing. There was also concern about the negative impact of restrictions on prenatal care quality and experience.

**Conclusions:**

This study demonstrates the impact of visitor restrictions on patients' prenatal care experience and perception of health care quality during the COVID-19 pandemic. Future public health strategies should be individualized to different patient populations addressing knowledge, health literacy, and socioeconomic status, and developed in conjunction with pregnant patients as key stakeholders in the delivery of prenatal health care.

## Introduction

The COVID-19 pandemic initiated a cascade of policies and procedures to control the spread of SARS-CoV-2 among patients, health care providers, and communities. Visitor restrictions were an essential component of these strategies.^1,2^ These policies entailed limiting the number of individuals who could visit a patient admitted into the hospital or accompany a patient to an in-person visit, and ranged in number based on the wave of the pandemic and individual institutional policies. Implementing strategies such as visitor restrictions in health care settings during an infectious public health threat is not a new approach.^3–5^

Studies show that hospital visitors had the potential to be a major contributing factor in infectious disease outbreaks.^6^ For this reason, these types of restrictions were one of the first strategies employed during the COVID-19 pandemic. While these types of restrictions were critical for the management of all patients, these strategies had a significant role in the care of pregnant patients, a population for which very little data were available in the early stages of the pandemic about the impact of COVID-19 for maternal and neonatal outcomes and evidence-based mechanisms to prevent infection and manage those patients with infection.^7–11^

A growing body of literature on the impact of visitor restrictions brought attention to the significant and often tragic impact of these policies on patients and families. The majority of these conversations focused on restrictions implemented at acute care units, skilled nursing facilities, and the management of specific patient populations.^6^ These studies demonstrated the negative impact of separation for patients and families, despite the benefits of preventing infection.^6^ Dramatic changes in visitation policies and communication practices may do more harm than good, specifically, modifications which had major implications for patients, family members, and health care at large.^12,13^

Evidence from past studies have confirmed that present family or support persons are central in the delivery of patient-centered health care.^13^ Yet, the ramifications of visitor restrictions in the prenatal care setting have remained relatively unexplored.^14–20^ Studies conducted before the COVID-19 pandemic showed that many pregnant patients rely on a support person to navigate these different situations, such as a spouse, partner, family member, or friend.^21–26^ Thus, studies of these restrictions on pregnant patients and their support system are essential in understanding the complete scope of the impact of COVID-19 on health care delivery.

Initial attention focused on patients' experiences on labor and delivery, an essential part of health care quality, safety, and patient experience.^14–16^ However, there has been a lack of data about the outpatient setting where the majority of prenatal care takes place.^27,28^ This is a clinical environment that may be seen as less acute, but bares the potential for a sudden change in obstetric outcomes (*e.g.*, the detection of pregnancy loss, detection of an anatomical abnormality). Given current gaps in the literature, we conducted a study to investigate the effects of visitor restrictions on pregnant patients and the delivery of prenatal care.

## Methods

All research procedures were approved by the Cleveland Clinic Institutional Review Board. Participants were 18 years of age or older, were English speaking, had a viable intrauterine pregnancy, and received outpatient obstetric care through Cleveland Clinic Health care System. Recruitment took place between May and July 2020. This is when visitor restrictions fluctuated between zero and/or one allowed visitor in the prenatal outpatient setting. Recruitment was structured to seek input from two groups of patients in the first trimester of pregnancy (Group 1) and patients in the second trimester (Group 2). All the participants from G2 and the majority of participants from G1 had been pregnant at the declaration of the pandemic in Ohio (March 2020), a time in which the most restrictive policies were in place. Recruitment was continued until thematic saturation was reached.

Participants were interviewed using a semi-structured interview guide. The interview guide was developed by content experts in obstetrics, infectious disease, COVID-19 policy development, medical decision-making, health care communication, and ethics. Then, the interview guide was field tested by the research team. All interviews were digitally recorded and transcribed verbatim with quality review to ensure the accuracy of the transcription.

Data analysis for these interviews was conducted as an iterative and progressive process of data immersion, coding, creating process memos, and thematic analysis, an approach consistent with grounded theory.^29,30^ We identified content domains and categories within the transcriptions to create a coding tree that was used to organize the data. A companion codebook was created to serve as a reference for the analysis. The transcripts and coding tree were uploaded to NVivo (version 12).

The research team held weekly meetings to review data coding and memos to identify themes. Themes that were identified were contextualized with information about the trimester of pregnancy, gravity/parity, and previous pregnancy experiences. Demographic and reproductive data were utilized to clarify findings and how they related to these variables. The research team made special note of those patients who have had prior pregnancies, those for whom this was their first pregnancy, and those with past telehealth experiences (Table 1).

**Table 1.** Demographics

Demographics of participants	Total (***n*** = 40), ***n*** (%)
Age	32.25 ± 4.54
Non-AMA (<35)	27 (67.5)
AMA (≥35)	13 (32.5)
Race	
White	34 (85.0)
Black	4 (10)
Asian	2 (5.0)
Reproductive history	
Primigravida	15 (37.5)
Multigravida	25 (62.5)
Trimester of pregnancy	
First trimester	20 (50)
Second trimester	20 (50)
Experience with telehealth before the current pregnancy	
Yes	6 (15)
No	34 (85)
Experience with telehealth during the current pregnancy	
Yes	24 (60)
No	16 (40)

AMA, advanced maternal age.

## Results

Thematic saturation, the point at which no new themes were identified, occurred at 40 interviews. Twenty patients were in Group 1 and 20 were in Group 2. The average age of participants was 32 years (±4 years standard deviation) (Table 1). Qualitative analysis identified three primary themes of (1) increased maternal concern, anxiety, and mental health concerns stemming from the lack of in-person partner support, (2) disappointment and lost experiences for patients seeking support from their partner during pregnancy, and (3) impact on prenatal care quality and patient experience (see Table 2 for supplemental data).

**Table 2.** Themes with Illustrative Quotes

Theme	Illustrative quotes
Increased maternal concern, anxiety, and mental health	“When I went for my 20 week anatomy scan, while I was waiting for the doctor to come in, I had a little moment of panic like, ‘What if something is wrong and I am by myself? I have to hear this information by myself?’ That was unnerving.” (G2–03) “With my profession, knowing about all the defects that could potentially happen and being more fearful of those and my husband not being able to attend with me, it has kind of that extra stress factor that, if something were to happen, I'd be there by myself. Luckily, everything has worked out well, and I understand all the precautions and the necessity. It's just higher stress levels going into that appointment.” (G2–02) “I've gone to all my appointments but I always have to go alone. I don't get to bring like my mom with me or my husband or somebody that can be there to support me in case, God forbid, I did get some kind of bad news or something like that.” (G2–18) “I had a miscarriage [referring to a prior pregnancy], and, if I'd been at my first appointment and found that news out, that would have been very different being by myself.” (G2–21) “The nuchal translucency and the blood draw, the [deidentified cfDNA screen], for that I was still pretty nervous about the health of the fetus. I was more worried about finding out by myself.” (G2–23) “I mean I think overall there's a lot of disadvantages to being pregnant during the coronavirus. Just from the point of a lot of times you feel like you're going through it by yourself. Because, like with my last baby, my husband could be by my side and everything and now it's like you kind of…you're already carrying the baby on your own but having your significant other not being able to go to appointments with you or ultrasounds … I just felt a little more isolated. I guess because you can't do as much together … I have a lot of concern about what kind of changes hopefully the medical field will be able to employ and make it so people don't have to go through some of these experiences alone. It can be kind of scary.” (G1–18)
Disappointment and lost experiences for the patient during pregnancy, seeking support from her partner during pregnancy	“That and a sense of wanting to feel more attachment because he was so involved with the first one and not the second one.” (G1–02) “I was upset when I found my husband could not come to appointments with me. So, that was definitely the first time I felt that the virus was really affecting something on a bigger scale for me.” (G1–09) “Then I'd say the biggest thing is that my husband is not able to go with me to my appointments especially being our first child. It really sucks. I was able to video chat with him but it's not obviously quite the same.” (G1–11) “I think the obvious downside was for the first ultrasound. My husband wasn't allowed to come in … I was still able to get care, but it was that emotional impact that he couldn't be there to share those moments” (G2–07) “I guess the negative of that is that I have to go alone. I'm not able to have my husband there. For our previous pregnancy he was there at every single one. So, that has been a little bit hard to kind of just have to be by myself looking at the progress.” (G2–09) “The only thing that was kind of a bummer is my husband couldn't come to my ultrasound appointments. I was able to FaceTime him which was nice, but, you know, not exactly the same thing so.” (G2–15) “In my previous pregnancies they [prior partners] weren't really involved like that. So, they didn't go to the ultrasounds. They didn't care about the OB visits, stuff like that. And my current partner does, and he wants to be there and he wants to do all that. So, I had the opportunity to actually experience it with somebody for the first time but it got taken from me because of COVID.” (G2–17)
Impact on prenatal care quality and experience	“He [her partner] has to depend on me to keep him updated with the baby. He wants to be there, be able to ask questions, which I totally understand” (G1–05) “I think it's important to have a person involved as a support system and who is involved in the decision, hearing right from the horse's mouth kind of what's going on” (G1–13) “I go to the doctor by myself … I am having to relay that information to him [her partner] now since he can't go” (G1–02) “When I was at my appointments, because my husband couldn't be with me, we couldn't have the conversations there with our provider to kind of make that decision [about prenatal genetic testing]. So, with the [deidentified cf DNA screen], I asked a bunch of questions and then I came back and talked to my husband and you know made sure that, ‘Okay, I don't have to get it now right? You can put an order in aside to get it later, you know, once I have this conversation? That's okay, right?’ And, maybe had he been in the office with me, I would have made a decision right then and there” (G2–15) “I believe that it's impacted my care in the fact that I can't have the support system that I feel that I need.” (G1–17) “There is not much context from across the room. It feels a little more concerning. I feel with my last pregnancy, my office and everything was on a personal level. I loved them. Now, it's like, ‘Oh, I have to go to the doctor.” (G1–05)

### Increased maternal concern, anxiety, and mental health

Participants reported heightened concern and anxiety from the lack of support person present with them during their prenatal care, particularly in situations where an obstetric complication could quickly occur or be discovered. This theme pertained to considerations about the actual absence of their designated primary support person or anticipated fear of this becoming a reality during their pregnancy. Reactions ranged from “increased fear” and “scary” to “much more anxiety.”

Most participants focused their concerns on significant landmarks in outpatient prenatal care, such as sonograms to identify fetal cardiac motion or to conduct an anatomy scan. For others, every outpatient visit marked a significant milestone, for example, when they could hear or see the fetal cardiac motion as confirmation of viability. As described by this participant, “Not being able to have my husband with me at my prenatal appointments has been horrible. Going to the ultrasounds by myself, going to the doctor's appointments by myself, it's just been awful. Because I'm just so afraid that something is going to happen and I'm going to be all alone” (G1–17).

This fear was expressed by those who had a prior pregnancy and those for whom this was their first experience with prenatal care. Current or prior obstetric complications compounded their concern: “With the fact that I've had so many abnormal tests come back… it's like I'm sitting in the office after I've had the ultrasound done, you know, crying by myself because my significant other couldn't be with me” (G2–17).

While some participants were able to use their mobile device to try to seek support, while in the exam or ultrasound room during these times, it was not seen as an acceptable substitute, either because of issues with the technology (*e.g.*, internet or mobile access overall or in the medical facility) or because of the lack of in-person contact (*e.g.*, holding hands) when the health care provider shared concerning news. This participant explained, “I got to Facetime him a little bit during, but the signal was horrible. So, it didn't work out that well” (G2–02).

A sense of separation from their partner during critical moments in prenatal care raised concerns about mental health. For this participant, the discussion about the absence of her partner at visits evolved into a larger discussion about concerns for postpartum depression:

“There's this huge emphasis on protecting everybody because of the virus but there's also… I think that we will find this out as time goes on… psychologically there has been probably a lot of damage done to people who have had adverse mental and psychological effects because of it [the pandemic]. You know and not just saying people who are can't go anywhere and are stuck inside quarantined. Obviously that can be difficult. But, like for myself somebody that needs to get medical care during the time, and for something for me…it's my first pregnancy and I can't take that back, I can't redo it. It's a one-time thing. It's affected my ability to have my husband be there with me for appointments that I'll never get back and there's no like doing it later or anything like that. I guess it's just been- for me that been the hardest thing and the most concerning thing about the virus is how is it going to impact my entire pregnancy, how's it going to impact my birth, how's it going to impact, you know, who I can have with me at my birth” (G2–18).

### Disappointment and lost experiences for patients seeking support from their partner during pregnancy

Participants reflected on the nature of these losses for themselves, their partner, and their relationship experience as a result of visitor restrictions. As described by this participant, “I'm sure if it was my first and my husband's first together, it would be a very different story because you want to experience all of that together. It's sort of part of the pregnancy process that you are used to or you kind of expect. Like you're really excited for the ultrasound and you want someone there to share in it with you” (G2–21).

Participants spoke of their partner feeling “left out” of the prenatal experience, particularly “hallmark moments” of the pregnancy. As described by this participant who expressed two levels of sadness: one for her own sense of a lost experience and also for that of her partner, “I am a little bit sad that my husband has not been able to come to the ultrasound appointments and the OB appointments …I know it makes him sad as well … I feel like he's getting cheated. We haven't decided if we are going to have a second child, and I keep telling him like I need to get through the first one first and see how this goes. And if we don't conceive again, he's sort of cheated out of the experience. So that, I feel bad for him” (G2–06).

While participants spoke of the loss of an ideal experience in which they could share this experience with their partner or support person, they also discussed the impact this had on the support they expected during pregnancy and the potential implications for their partner's attachment to the pregnancy and child. One participant captured this concern as, “I didn't necessarily feel like my husband has been as involved this pregnancy as he was [with] my daughter. You know, with my daughter, he was able to attend my first appointment and at all of my ultrasounds. So, he got to, with my first pregnancy, he got to see, you know, the baby move on the ultrasound screen. He hasn't been able to do that this time … For him, it's been different because he hasn't been able to really see anything. He hasn't been able to hear our baby's heartbeat like it was before or anything” (G2–16).

Some participants wanted to support their partner's having a sense of agency in the pregnancy as a shared experience in both routine care and during moments when a complication was detected. As described by this participant, it was not just a matter of a lost experience for the couple but also the partners' ability to support her in a way that allowed him to feel that he had an active role to play in the pregnancy: “I think it kind of takes away from our first pregnancy and the experience of us doing this together. And I was worried, if I went to an appointment and something was wrong, he wouldn't be by my side” (G2–11). For some, the partner's absence diminished the support they hoped to establish in moments that made the pregnancy “real” for them.

This participant reflected on her concerns about what her partner's absence may mean for the support she could expect from him during pregnancy and his relationship with the child after birth. “I know that to me, that's [learning about the sex of the fetus at the time of the ultrasound] the biggest sharing point of when they find out if it's a boy or a girl, because that's when they kind of be more invested because that's when they can call it something instead of just ‘baby’. So, I could see that really impacting the relationship of the dad with the preborn baby. Because when you find the gender[sic], a gender is a person. For a lot of guys, before that, it's just nothing” (G1–13).

For many, a sense of isolation emerged due to situations in which their partner could not be present in person. Participants talked about the sense of isolation that began in practices to social distance compounded by a sense of being alone during their visits. As described by this participant, “I also have learned to get over it, but it still kind of gets to me sometimes when I have to go to appointments by myself. I just knew going into this pregnancy that my partner would be with me the whole way whether I got good news or bad news, and that whole things upsets me that I have to, like I'm not in it alone, but, in that moment, I am alone, and it's just me and doctor” (G1–03).

### Impact on prenatal care quality and patient experience

Several participants described how visitor restrictions extended to their perceptions of the quality and experience of their prenatal care. One aspect included concerns about accessing information about prenatal care and making decisions in an informed manner, which met their and their partners' decision-making needs. In addition, some expressed concerns about potential delays in the context of visitor restrictions that might occur if the partner was not present and no advanced decision-making had taken place.

This participant reflected on her ability to accurately convey information about her prenatal care choices to her partner, “I have to relay all the information back to him from the doctor. So, I'm in the appointment scribbling notes and trying to have all the questions to ask the doctor so he also has the same information as me about our pregnancy… I think he would prefer to be there and have those conversations as well like together” (G2–20). This included real and perceived barriers for a patient asking questions and seeking information that the partner may have wanted or questions that the patient may not have otherwise asked: “I'm sure he [her partner] would have questions that I do not even have or consider” (G2–06).

For this participant, the tenor of the visit changed without her partner, which altered the patient-provider dynamic during the visit: “Not being able to have my partner there was a little intimidating” (G1–10).

Patient experience was also affected. Participants described their prenatal care experience as “disappointing” and caused “increased anxiety” during the pandemic. While this participant was grateful to access care during the pandemic, the care she received was not what she had expected: “We are still receiving quality care one way or another, but I think the experience itself is different. Like, not being able to have a family member, spouse, or if someone has a doula or something to attend their appointments with them. They can't do that so, it's like no support, at least while you are there at the appointment” (G1–20).

Other participants questioned the rationale behind how visitor restrictions were developed for obstetric care and the larger implications of pandemic precautions for patients. As described by this participant, “My big concern with all this is that healthcare providers and people are so afraid of the virus they're losing touch with basic care of things that they should be providing regardless of people's COVID status” (G2–10).

## Discussion

This study sheds light on the important repercussions of visitor restrictions in prenatal care. Participants in this study described their preferences to have a support person available and in-person during their prenatal care, ranging from a domestic partner, co-parent, or family member to a friend who they desired to be part of prenatal care decision-making. This included the need to have a partner present to help navigate some of the complex discussions with their health care provider and decisions in prenatal care. It was also seen as an opportunity to forge a shared experience during pregnancy to manage both positive and negative outcomes during the pregnancy, with enduring benefits of this strengthened relationship after the child was born.^21–31^

Studies conducted before the COVID-19 pandemic describe these similar roles for a support person and patients' preferences for a partner to be present, if desired or available, during key times of their prenatal care. That may include specific gestational age-based time points in obstetric care (*e.g.*, second trimester anatomical sonogram, decisions about prenatal genetic screening and diagnostic testing), in addition to the routine activities that take place during the majority of outpatient visits (*e.g.*, auscultating the fetal heartbeat).^21–31^

Participants also discussed how the uncertainties and stressors of the pandemic augmented the preference to have an in-person support partner and, in turn, the impact of visitor restrictions limiting this resource. Immediate and short-term psychosocial concerns were cited, including increased fear from being alone during a visit when a potential complication was suspected or confirmed. While many participants reflected on their own experiences with being separated from their support person, others were deeply concerned by the perceived threat of visitor restrictions, particularly as health care systems rapidly adapted their policies in response to the severity of the pandemic. Ramifications for health care quality and informed decision-making were also noted.

Participants who would have wanted to have a support person with them spoke of concerns about the ability to make the kinds of decisions about their prenatal care (*e.g.*, the decision to initiate prenatal genetic testing), which they felt would reflect the needs and interests of their partner. There was also the preference to have a partner present to help guide conversations about complex and value-based decisions such as prenatal genetic screening and diagnostic testing, asking questions and partnering to achieve a shared decision-making process to obtain information that met their needs as individuals and as a family.^21–31^

However, due to visitor restrictions in the outpatient setting, there was a concern about the ability to make a shared and informed prenatal health care decision. While video meeting formats (*e.g.*, Zoom, Facetime) assisted some who could access it, others felt that the support they needed could only be achieved with their support person present, standing by their side, holding their hand, or providing other in-person comforts.

These responses are significant to note, given the degree of emotional reactions and worry documented in studies conducted before the COVID-19 pandemic about the experiences and responses of patients who experienced a pregnancy complication or loss, with emotional, psychological, and social effects often exacerbated by prior reproductive history (*e.g.*, prior history of infertility, miscarriage, or intrauterine fetal demise). Studies also shed light on the role of a partner as a source of support during these times.^32,33^ Our study findings highlight the additive stressors that visitor restrictions may have placed on pregnant patients, including those who experienced a prior obstetric complication, who had a fear of such an outcome during the current pregnancy, or who were unprepared to attend a prenatal care visit alone.

There were also concerns about the long-term ramifications of visitor restrictions experienced in the outpatient setting. Family relationships were at the core of some of these concerns. These fears pertained to how a partner may bond with the child after birth and how their relationship with the partner may fare without the opportunity for a shared experience of the pregnancy. Studies show the multiple benefits that pregnant patients may seek from having a partner present at the outpatient visit, including providing an opportunity for the partner to develop an emotional connection with the pregnancy and the pregnant person.^34,35^

There were heightened concern about mental health, emphasizing possible increases in the incidence and severity of postpartum depression among pregnant patients during the pandemic. Participants reflected on the sense of isolation, founded in separation as a result of social distancing, and then compounded by attending prenatal care visits without a partner present in person.

While all study participants were pregnant at the time of the study, they reflected on concerns that may affect them at a later time during the pregnancy and in the postpartum period. For example, participants reflected on concerns about maternal mental health and postpartum depression.^36–39^ The significance for maternal and infant wellbeing, in addition to an increasing rate of postpartum depression before the COVID-19 was well documented, with emerging studies demonstrating increased risk as a result of the events of the pandemic.^20,40–44^ What these rates mean for long-term mental health and depression affecting future pregnancies is unknown, but must be understood. In doing so, it will be important to understand how the entire prenatal care experience, not just the significant events in the inpatient setting, may impact patients' mental health and wellbeing.

This study highlights the potentially significant impact of visitor restrictions in an outpatient care setting. Combined with emerging data about the effects of visitor restrictions on patients during labor and delivery and postpartum health, it is critical that attention is paid on how best to manage infection control interventions during a public health crisis such as the COVID-19 pandemic. Video conferencing platforms for telehealth and patient-family communication have been a vital mechanism to deliver health care during this pandemic.^45–47^ However, such technologies may not be an equitable strategy to support health care and patient experience. The pandemic amplified the digital divide noted among populations who could not access the technology or internet to utilize telehealth or other internet-based modalities to support communication.^44,48–52^

Furthermore, it may not be an acceptable alternative for patients awaiting news of fetal viability or health, who seek and expect a specific level of patient experience during pregnancy. Given these emerging data, we propose that future visitor restrictions should be developed in partnership with patients and their support partners as key stakeholders in the implications of such policies. In addition, we propose that health care providers develop scripting to provide empathetic responses to patients whose partners cannot be with them due to visitor restrictions. Furthermore, practices should develop resources for patients when they seek to engage a partner who cannot be present in prenatal care decision-making. Furthermore, rigorous studies and metrics are required to fully understand the long-term ramifications of these policies during the pandemic.

While our study illuminates the emerging challenges acquired with visitor restrictions during the pandemic, some limitations were considered. The study utilized qualitative methods among a sample of patients at a single health care system in Ohio, acknowledging that local policies at other health care systems in the nation may have had different levels of visitor restrictions for obstetric care. While our sample represented patients of different reproductive histories, our sample was limited in racial and ethnic representation.

In addition, our study is limited to the views of those who had a support person involved at some time in their care, a term that we defined broadly in our study procedures as a domestic partner, co-parent, family member, friend, or other individual who the participant invited to be part of prenatal care decision-making. Thus, the perspectives of those without a decision support partner are not reflected in this study, calling for the need to include this population in future research. Despite these limitations, the study brings to light important findings for which further research is needed to elucidate.

## Conclusions

This study demonstrates the impact of visitor restrictions implemented during the COVID-19 pandemic. While limiting visitors certainly affects labor and delivery, this study shows the effect these policies have on patients in the outpatient setting. Solutions proposed and implemented during the pandemic, such as video conferencing, may not adequately address the needs of pregnant patients during this unique context of health care delivery or provide a reliable and equitable mode of health care. It is essential that visitor restriction policies during the COVID-19 pandemic or in other future public health crises are developed in conjunction with pregnant patients and their family members as key stakeholders in obstetric care and the wellbeing of children and families after birth.
